# Microdynamic flowability for early API characterisation: A case study on Palbociclib

**DOI:** 10.1016/j.pscia.2025.100069

**Published:** 2025-03-14

**Authors:** David Blanco, Nicolas Pätzmann, Pablo García-Triñanes

**Affiliations:** a Division of Pharmaceutical Chemistry and Technology, University of Helsinki, Finland; b School of Pharmacy, University College Cork, Cork, Ireland; c Department of Chemical Engineering and Food Technology, Faculty of Sciences, University of Cadiz, 11510, Puerto Real, Spain

**Keywords:** API characterisation, Microdynamic powder flowability, Micronisation, Pre-formulation, Particle engineering

## Abstract

This study explores microdynamic flowability as an innovative approach for early active pharmaceutical ingredient (API) characterisation, when compounds are often scarce and/or expensive. By incorporating small-scale flow measurements during the pre-formulation stage, we aim to support strategic decision-making in formulation development and process design. Laboratory-scale micronisation of the poorly water-soluble drug Palbociclib, while enhancing dissolution, was found to adversely affect flowability. Agglomeration driven by cohesive forces was quantitatively described for the first time via image analysis using sample quantities of less than 200 mg. Our findings demonstrate that microdynamic flow studies provide critical insights into the processability of APIs under low-stress conditions, such as those relevant to research and development (R&D) tablet presses. These results highlight the value of advanced flowability analysis in early-stage development, enabling improved understanding and control of powder processing in pharmaceutical manufacturing and particle engineering.

## Highlights

• Powder flow measurements are integrated into the earliest stages of drug development.
• Pre-formulation flow data relevant to R&D tablet presses can now be generated.
• Micronisation enhances dissolution but worsens flowability due to agglomeration.
• Advanced image analysis enables quantitative detection of powder agglomeration.
• Microdynamic flow studies aid early formulation development and process design.

## Graphical abstract

**Figure:**
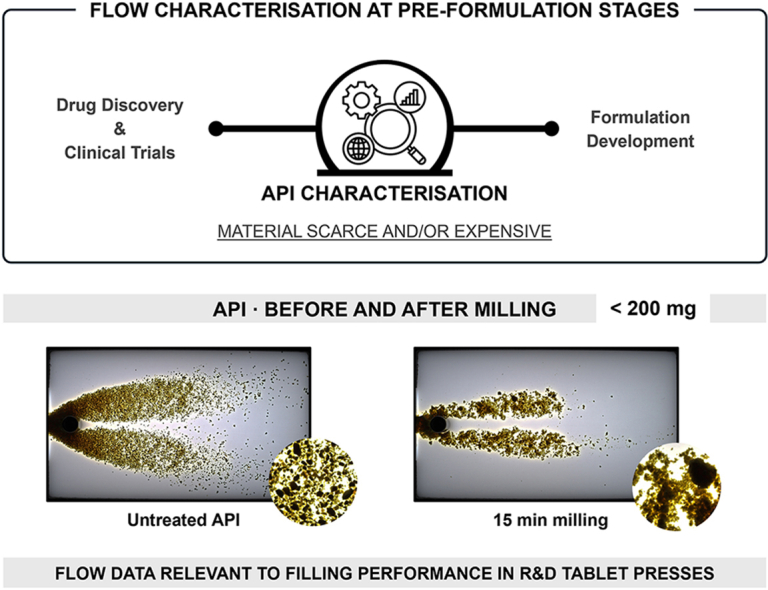

## 1 Introduction

Drug research and development is a complex process that often takes several years from the discovery phase to commercialisation. After testing and screening a new chemical entity (NCE) through the process of drug discovery and confirmation of its efficacy and safety in clinical trials, the selected drug candidate, commonly known as an active pharmaceutical ingredient (API), needs to be formulated and scaled up to serial production. At the beginning of small-scale R&D, generally referred to as the pre-formulation phase prior to clinical trials, the API is extensively characterised to define the most suitable manufacturing route (i.e. the pertinent unit operations) and to support excipient selection [1]. This includes various investigations, such as physicochemical analysis, biopharmaceutical profiles, compression trials and flowability assessments.

For efficient manufacturing, an API should exhibit good flowability (i.e. it has a low tendency to agglomerate) and compact efficiently. However, this does not normally happen, and APIs typically require processing before tableting or encapsulation. Additionally, many new APIs have poor solubility, thus requiring particle size reduction techniques to improve dissolution and bioavailability, a process that often compromises powder flow [3,18]. Regardless of the manufacturing route, successful processing depends on filling consistency, as it directly affects the uniformity of the dosage form (mass variation and drug content), as well as subsequent compaction and ejection steps, ultimately influencing tablet quality attributes [10,14,21]. While compaction simulators effectively characterise compression properties using small material quantities, they do not assess pre-compression events, such as hopper discharge, die filling, or powder segregation over time [19].

If the material is scarce, as is often the case in small-scale formulation development and early API characterisation, powder flow analysis is often not conducted. Most traditional testers in industry require tens of grams for measurement [2]. Previous work introduced microdynamic flowability as a novel approach to characterise flow properties using very small amounts of powder sample (less than 200 ​mg). Early assessment of die-filling performance was achieved from the beginning of the formulation work and the focus was shifted from end-product testing to building quality throughout the development process. Current flow testing methods based on bulk measurements failed to characterise the processing conditions in small-scale R&D tablet presses (i.e. dynamic powder flow properties at the very low stress range) [4,5]. The reality is that powder flow analysis remains largely inaccessible in the early stages of development.

This study introduces microdynamic flowability as a strategic tool for assessing API processability from earliest product development. By introducing small-scale measurements, we aim to bridge the gap between material characterisation at pre-formulation stage and real-world manufacturing performance. To achieve this, we analysed flow data relevant to the filling process for the model drug Palbociclib before and after milling. Our findings provide insights into the early-stage formulation strategies necessary for optimising solid dosage manufacturing. Given that consistent and reliable flow during die-filling is crucial to manufacturing costs, time-to-market and final product quality [11,16,22], this study highlights the importance of advanced flowability assessments in pharmaceutical development.

## 2 Materials and methods

### 2.1 Materials

Traditional mechanical milling was applied to a BCS Class II (poor water-soluble) drug candidate, Palbociclib (MSN Pharmaceuticals Inc., New Jersey, USA), which requires to be processed to enhance dissolution and bioavailability. As a cornerstone therapy for advanced or metastatic breast cancer, its high cost and limited availability during early-stage development further highlight the importance of emerging powder flow analytical techniques that minimise sample consumption. Untreated API was used as reference to evaluate the effect of milling on flow and dissolution properties.

Standard microcrystalline cellulose (MCC) grades, including Vivapur® 101 (Ph. Eur grade, JRS Pharma, Rosenberg, Germany), Avicel® PH-102 (Ph. Eur grade, FMC BioPolymer, Ireland), and Avicel® PH-200 (Ph. Eur grade, JRS Pharma, Rosenberg, Germany), were employed as reference materials to rank the API's flow behaviour. These MCC grades span a spectrum of flow properties typically encountered in die-filling processes during pharmaceutical manufacturing. Their inclusion provided a comprehensive framework for benchmarking and validation, since traditional bulk flow measuring techniques could not be applied in this study.

### 2.2 Physical characterisation

Particle size and size distribution (PSD) were determined by laser diffractometry (Mastersizer 2000 with Hydro 2000S, Malvern Instruments Co. Ltd., Solihull, UK). Given the high toxicity of Palbociclib in dry conditions and the substantial sample requirement (over 2 ​g), the wet dispersion method was chosen. The drug powder (200 ​mg) was added to 50 ​mL of water and 0.05 ​mL of Tween 80. To prepare a dispersion, the sample was stirred and mixed for 10 ​min (±30 ​s) and then immediately placed in the diffractometer until a stable obscuration between 10 and 20 ​% was achieved. The refractive index for the dispersion medium (water) was set to 1.33, and the value for the particle was selected based on published refractive indices of the respective compound. The stirring speed was set to 2100 ​rpm, and the measurement duration was 30 ​s. All measurements were carried out in triplicate and average values were calculated.

Particle morphology and surface microstructure were examined using scanning electron microscopy SEM (Quanta FEG250 SEM, ThermoFisher, Oregon, USA) equipped with a large field low vacuum detector [LFD]). Powder samples were sputtered with gold prior to the examination. SEM measurements were performed at 15 ​kV with a beam current of 2 ​× ​10^−10^ A and the distance to the sample was set at 15 ​mm to achieve good image quality.

### 2.3 Micronisation and dissolution studies

Milling was conducted using a vibrational ball mill (MM 200, Retsch, Haan, Germany). 250 ​mg (±0.1 ​mg) of the model drug and three 9 ​mm stainless steel balls were placed in a 25 ​mL stainless steel milling jar. The milling process lasted 15 ​min at a frequency of 15 ​Hz. Dissolution was tested in a USP2 vessel with a stirring speed of 50 ​rpm in 250 ​mL FaSSIF at 37 ​°C. The dissolution experiments, lasting 80 ​min, were monitored using fibre-optic UV probes connected to the Rainbow Dynamic Dissolution Monitor Instrument (Pion Inc., Billerica, MA, USA). Standard curves were established using the second derivative of the UV absorbance. Freshly prepared samples (120 ​± ​30 ​min after preparation) were added to the medium in triplicate.

### 2.4 Microdynamic flow studies

**Fig. 1:**
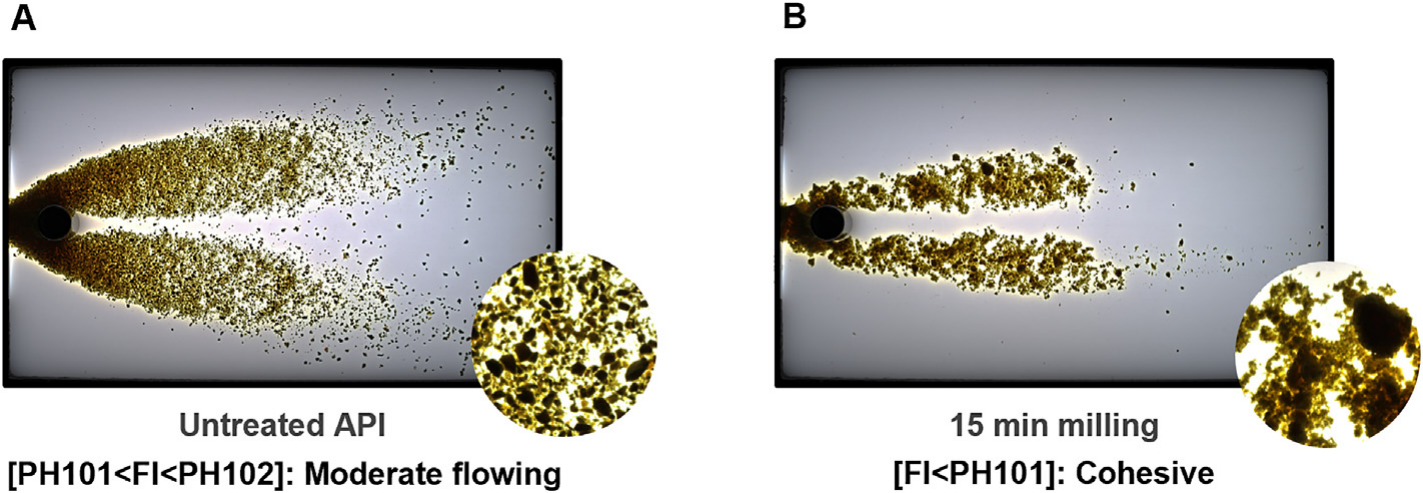
Displays microdynamic flow patterns before (A) and after milling (B), visually illustrating the increased cohesion (or agglomeration) in the milled sample. The zoomed-in regions are presented at the same magnification for both samples to facilitate cross-comparison.

Miniaturisation of pharmacopoeial powder flow through an orifice employs patented technology to measure dynamic flow properties on a small scale (<200 ​mg) [8,20]. A precisely controlled mechanical stress (linear motion and related dynamics) is applied to discharge the powder sample through an orifice, replacing gravity in conventional hooper discharge techniques. No additional stresses are applied to the measurement. A scheme of the measuring system and testing setup, including a detailed diagram of the measuring cuvette and the powder flow inside, can be found elsewhere [4]. The resistance of the powder to such displacement yields unique flow patterns, depending on material properties, as shown in Fig. 1. The flow behaviour is inferred from observable changes in the system via image analysis. A three-step conditioning process ensures a reproducible initial packing state for a fixed powder volume (0.2 ​cm^3^).

The horizontally positioned measurement setup enables advanced imaging of powder flow properties. MIPAR software (MIPAR Image Analysis, LLC, Worthington, OH, USA) was used for image processing and particle characterisation to ensure accurate segmentation and data extraction. Image processing defined flowability using the Flow Index (FI, %), calculated as the area fraction or percentage of pixels occupied by the powder after flowing through the orifice, relative to the total illuminated background area. Higher FI values indicate better flow properties as lower shear stresses counteract the applied dynamics (i.e. lower cohesion within the powder mass), as shown in our previous publications [[4], [5], [6]]. Three grades of microcrystalline cellulose common pharmaceutical applications (MCC) –PH-200, PH-102, and PH-101 (Ph.Eur. grade)– with distinct flow properties were selected here as model materials to assess and rank flowability data (Table 1). Medium MCC grade PH102 was considered at the boundary between an acceptable and a poor flowing excipient for high-speed machinery, as generally accepted in powder technology [21]. It is noteworthy that the flow classification was conducted for a fixed flow rate (or applied dynamics), where all the model materials were conveniently classified in the novel measuring system.

**Table 1:** Microdynamic flow properties with model materials representing the boundaries for classification. The Flow Index (FI, %) is used to classify flowability, with higher values indicating better flow. Boundary materials are provided as references, where MCC PH-200 represents the best flow behaviour and MCC PH-101 the poorest. Intermediate flow categories are defined based on FI ranges between these materials.

FI, %	Flow Classification	Boundary Material
>50	Excellent	≫ MCC PH-200
30–50	Good	MCC PH-200 ≳ FI ​≳ ​MCC PH-102
10–30	Acceptable	MCC PH-102 ≳ FI ​≳ ​MCC PH-101
<10	Poor	≪ MCC PH-101

**Fig. 2:**
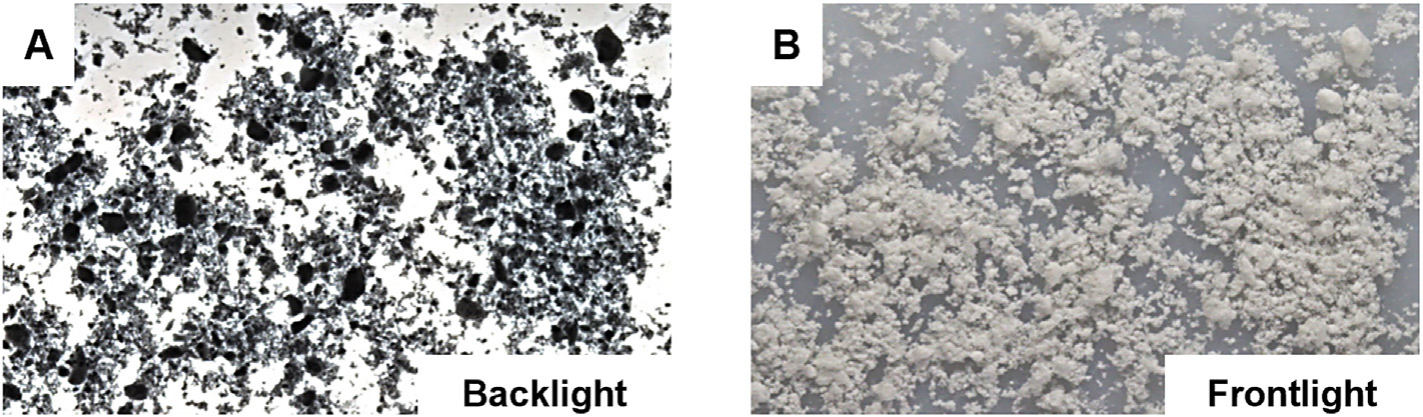
Illustrates agglomeration phenomena under different lighting conditions using the method: (A) Back lighting, (B) Front lighting.

Previous work introduced microdynamic flow properties to characterise material behaviour according to the filling performance in small-scale R&D tablet presses. Advanced agglomerate detection captured the mechanisms of flow enhancement and cohesion governing small-scale filling processes and that cannot be elucidated from numerical data. Agglomeration Ratio (AG, dimensionless, from 0 to 1 values) was therefore introduced in this study as an additional quantitative descriptor of powder flow properties resulting from the milling process. For cohesive materials, the AG described the cohesive forces acting in the particulate solid and was quantitatively determined from the area fraction or percentage of darker pixels within the powder flow pattern. For non-cohesive materials, the AG described darker coarse particles, which contrasted with the lighter fines fraction. An example of agglomeration phenomena due to cohesion is shown in Fig. 2.

#### 2.4.1 Optimised testing setup

To facilitate this analysis, the testing setup was optimised to include precise motion control, high-resolution digital imaging and interchangeable funnel geometries, providing maximum flexibility. This setup allows for the analysis of both coarser materials and more cohesive powders. For cohesive powders, where attractive cohesive forces exist between particles, these must be overcome to initiate flow. Higher breaking forces and wider funnel orifices facilitated the flowability of more cohesive powders, such as milled Palbociclib. Additionally, histogram analysis and Otsu's method were here applied to automatically isolate the darkest regions via thresholding. In practical terms, this approach divides the grayscale image into two major classes (cohesive agglomerates and individual particles) without requiring any manual input. Although Otsu's method is effective when the image histogram exhibits relatively bimodal distribution (e.g. distinctly darker cohesive lumps vs. brighter, smaller particles after milling), further refinement may be needed in cases where single, dense particles have similar grayscale values to those of agglomerates.

## 3 Results

### 3.1 Physical characterisation

**Fig. 3:**
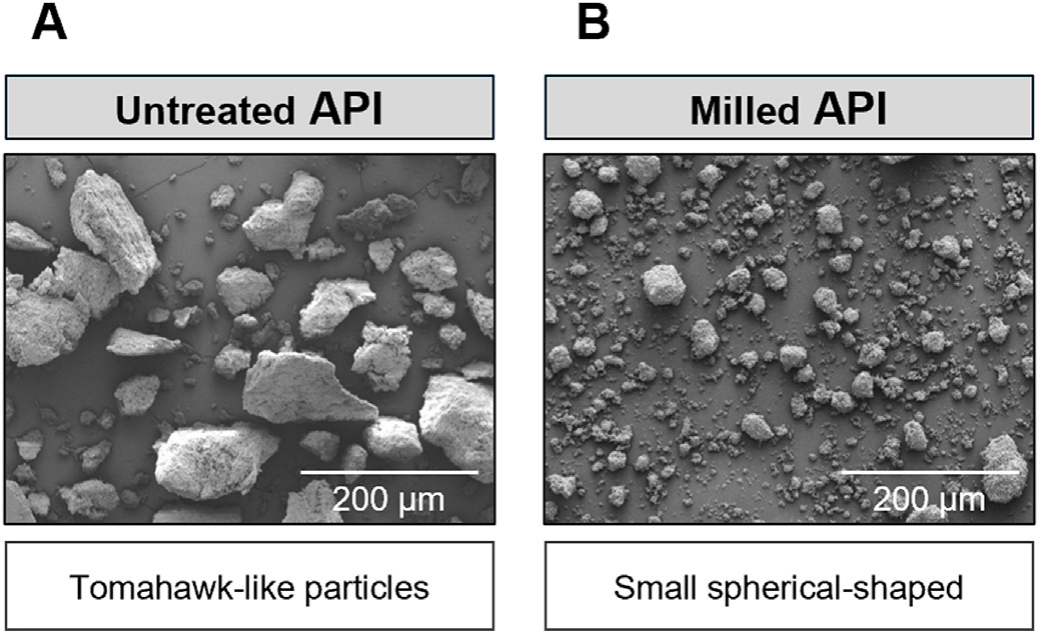
Presents SEM micrographs of Palbociclib before (A) and after milling (B), illustrating the morphological transformation due to micronisation. Both images are shown at the same magnification (× ​200), with a reference scale included.

Untreated Palbociclib showed as tomahawk-like particles. After milling, the API appeared as small spherical-shaped particles with a narrower PSD (Fig. 3 and Table 2). These observations confirm that milling significantly alters the particle size and morphology, which is expected to impact flow properties.

**Table 2:** Presents PSD data, highlighting the reduction in particle size and the corresponding span values, indicating a more uniform particle distribution after milling (*n=3*).

	Before milling	After milling
**Dv_10_** (μm)	21.86 ​± ​7.43	2.07 ​± ​0.11
**Dv_50_** (μm)	148.50 ​± ​16.26	38.74 ​± ​0.71
**Dv_90_** (μm)	556.65 ​± ​49.56	129.28 ​± ​7.92
**Span**	3.60	3.28

### 3.2 Effects of milling on flowability

Microdynamic flow studies described the effects on flowability of particle size reduction at a small laboratory scale (Fig. 1 and Table 3). A fixed flow (or shear) rate was used for cross-comparison and the same magnification was applied in the zoom-in regions for imaging consistency. These results demonstrated that micronisation significantly alters API flow properties, emphasizing the importance of early-stage flow characterisation in formulation development. The findings highlighted the need for strategies to mitigate cohesion-related flow issues during pre-formulation and manufacturing stages.

**Table 3:** Quantifies the flow index (FI) and agglomeration ratio (AG), showing a substantial decrease in FI (indicating worse flow performance) and an increase in AG (indicating higher cohesion) after milling (*n=3*).

	Before milling		After Milling	
	FI, %	AG	FI, %	AG
Palbociclib	28.35 ​± ​0.87	0.05 ​± ​0.01	15.52 ​± ​1.88	0.42 ​± ​0.03

## 4 Discussion

Dissolution profiles showed significant improvements after micronisation and increased specific surface area (results not included). For a detailed analysis of the effects of milling on dissolution profiles and material properties across various APIs, refer to Ref. [15]. From a flowability perspective, a narrower PSD and a smaller average particle size had an impact on flowability (Fig. 1). The spheroidisation of particles attributed to milling appeared to balance such micronisation and the sample exhibited moderately lower, rather than very poor, flow performance, consistent with previous studies on spherical starch particles [4]. Digital imaging was highly effective in visualising and interpreting these results. Image analysis detected agglomeration phenomena in the milled sample, driven by cohesive forces, such as van der Waals forces associated with particle size reduction, which hindered flowability. These findings highlight the challenges of maintaining technological processability after micronisation.

Recent advancements, such as small-sample predictive models and instruments like the SSSpinTester [7,12], have enabled the characterisation of fine cohesive powders using minimal sample quantities. While such approaches have shown good agreement with traditional shear cell testers, with differences less than 22% for cohesive materials, these predictions are validated against bulk flow behaviour, which may not accurately represent the very-low stress environments typically encountered during die-filling processes [[24], [25]]. For dynamic applications, none of these quasi-static test methods is predictive because they do not characterise the flow parameters at higher strain rates [9,13,17]. Consequently, while small-sample predictive models offer valuable insights, their applicability to scenarios involving very low stresses and/or high strain rates, such as during filling processes, remains limited and requires further validation. Further research is needed to evaluate the accuracy of these models under such specific conditions and to determine their potential as reliable substitutes for empirical testers in early-stage drug development.

### 4.1 Novelty and limitations

While previous studies primarily focused on microdynamic flowability in excipients and bulk powders [[4], [5], [6]], this study is the first to apply the method to a processed API (Palbociclib), demonstrating its potential for early-stage API characterisation. By introducing quantitative agglomerate detection, an advancement not achievable with existing flow testers, this research bridges the gap between early API characterisation and real-world manufacturing performance, ultimately supporting better formulation decisions. Leveraging image-based analysis, it provides deeper insight into powder cohesiveness and flow behaviour, which are critical for predicting die-filling performance and optimising pharmaceutical manufacturing.

A key advantage of this approach is its miniaturised, enclosed system, which enables powder flow measurements with minimal sample consumption (≤200 ​mg). This not only facilitates a safer handling of toxic compounds but also ensures feasibility when materials are scarce or expensive. Additionally, its non-invasive nature, operating at very low stresses via image analysis, preserves the physical and chemical integrity of the samples, allowing for their reuse in further analyses.

Despite these advantages, limitations exist when assessing highly cohesive APIs, particularly those with very small particle sizes that do not flow well under very low consolidation stresses. Microdynamic flow properties are relevant for filling performance in R&D tablet presses, where cohesive forces and agglomeration largely govern flow behaviour [4,5]. Higher cohesion leads to greater tablet mass variability, interrupted filling, and lower repeatability in the miniaturised method.

Differentiation of highly cohesive powders can be achieved using quasi-static methods such as shear cell analysis, which neglect the spatial (cohesive) distribution of the powder at lower stresses. However, these results are hardly extrapolated to dynamic applications such as filling processes, and require large sample quantities. For small-scale measurements in the quasi-static regime, further investigations with the SSSpinTester (Material Flow Solutions Inc.) are recommended.

### 4.2 Relevance and perspectives

Powder flowability is a critical factor in pharmaceutical manufacturing, affecting tablet mass variation, dosing accuracy and overall product quality. Highly cohesive APIs often present challenges in blending, die-filling and capsule filling, leading to inconsistencies, segregation and processing inefficiencies. Understanding agglomeration behaviour is essential for predicting manufacturing performance, as excessive cohesion can result in flow disruptions and formulation failures. The microdynamic flowability approach provides an early-stage, quantitative assessment of these factors, helping formulators identify potential flow issues from the earliest product development stages.

Depending on the API's manufacturing profile, a prototype formulation will eventually be developed for testing and, if validated, scaled up. The novel method can be applied at pre-formulation stages to define the most suitable manufacturing route and support excipient selection. Next, early assessment of filling performance during small-scale formulation development can support consistent product quality, minimise waste and reduce time-to-market [4,5]. Process-relevant flow data can now be generated from the lab to commercial production for enhanced understanding, monitoring and control of products and processes.

Future research should extend the applicability of this approach to other particle engineering techniques, such as co-crystallisation, granulation and spray drying. Additionally, integrating complementary techniques could provide a more comprehensive understanding of powder behaviour across formulation steps, ultimately enhancing the development of oral solid dosage forms (OSDs) and other powder-based formulations while reducing resource consumption. These advancements may also extend its utility to other industries, including food processing, additive manufacturing (AM) and chemicals.

## 5 Conclusions

Micronisation and milling techniques are routine aspects in pharmaceutical manufacturing, yet characterising API flow properties in early product development remains a challenge. In this study, particle size reduction of Palbociclib adversely affected flow properties at very low stresses, as observed in R&D tablet presses and quantified through microdynamic flow studies. This method presents key advantages: (i) it requires less than 200 ​mg of sample, enabling powder flow measurements relevant to R&D tablet presses at pre-formulation stages, (ii) it quantitatively describes agglomeration driven by cohesive forces through image-based analysis, and (iii) it employs a closed measuring system, preserving sample integrity and making it suitable for toxic compounds. Assessing API processability using this miniaturised approach can provide a cost-effective, sustainable strategy for early formulation development and process design, minimising waste and accelerating time-to-market.

## CRediT authorship contribution statement

**David Blanco:** Writing – original draft, Methodology, Investigation, Conceptualization. **Nicolas Pätzmann:** Methodology, Investigation, Conceptualization. **Pablo García-Triñanes:** Writing – review & editing, Resources.

## Data availability

Data supporting the findings of this study are available upon request from the corresponding author.

## Ethics approval

Not applicable.

## Declaration of generative AI in scientific writing

Not applicable.

## Funding information

This work was funded by the 10.13039/501100003125Finnish Cultural Foundation -Suomen Kulttuurirahasto Foundation- [2024] and the innovation program under the Marie Skłodowska-Curie (No. 778051, 2022). P.G.T was supported by a Beatriz Galindo Senior Fellowship from the Spanish 10.13039/100014440Ministry of Science, Innovation and Universities.

## Declaration of competing interest

The authors declare that they have no known competing financial interests or personal relationships that could have appeared to influence the work reported in this paper.
